# Effects of Chemosignals from Sad Tears and Postprandial Plasma on Appetite and Food Intake in Humans

**DOI:** 10.1371/journal.pone.0042352

**Published:** 2012-08-01

**Authors:** Tae Jung Oh, Min Young Kim, Kyong Soo Park, Young Min Cho

**Affiliations:** Department of Internal Medicine, Seoul National University College of Medicine, Seoul, Republic of Korea; Wageningen University, The Netherlands

## Abstract

Chemosignals from human body fluids may modulate biological functions in humans. The objective of this study was to examine whether chemosignals from human sad tears and postprandial plasma modulate appetite. We obtained fasting and postprandial plasma from male participants and sad tears and saline, which was trickled below the eyelids, from female volunteers. These samples were then randomly distributed to male participants to sniff with a band-aid containing 100 µl of each fluid on four consecutive days in a double-blind fashion. We checked appetite by a visual analogue scale (VAS) and food intake by measuring the consumption of a test meal. In addition, the serum levels of total testosterone and LH were measured. Twenty men (mean age 26.3±4.6 years) were enrolled in this study. They could not discriminate between the smell of fasting and postprandial plasma and the smell of sad tears and trickled saline. Appetite and the amount of food intake were not different between the groups. Although the VAS ratings of appetite correlated with the food intake upon sniffing fasting plasma, postprandial plasma, and trickled saline, there was no such correlation upon sniffing sad tears. In addition, the decrease in serum testosterone levels from the baseline was greater with sad tears than with the trickled saline (−28.6±3.3% vs. −14.0±5.2%; *P* = 0.019). These data suggest that chemosignals from human sad tears and postprandial plasma do not appear to reduce appetite and food intake. However, further studies are necessary to examine whether sad tears may alter the appetite-eating behavior relation.

## Introduction

Obesity is closely related to metabolic diseases, and its prevalence is sharply increasing [1]. However, the magnitude of weight loss by medical treatments is only modest [2], [3], and safety issues cast a dark shadow in the development of new antiobesity drugs [4]. Amid the repeated failures of many antiobesity drugs, which were mainly due to safety problems, there have been continued efforts to find new drugs for the treatment of obesity [5]. To curtail the constant threat of obesity, we need to find new ways to safely and effectively reduce food intake and/or to increase energy expenditure.

A pheromone is a secreted or excreted chemical factor that triggers a social response in members of the same species and is important in the social communication of a variety of species from insects to non-human primates [6], [7]. Pheromones regulate primitive behaviors in animals by functioning as primers, releasers, signalers, and modulators [7]. While the purported exploitation of pheromones by humans for social communication is still controversial [7], there are several lines of evidence demonstrating the presence of human pheromones or chemosignals. Human beings may have some primer pheromones, as evidenced by the menstrual synchrony observed in some women who share a common environment [8], [9]. Some mothers are able to recognize their babies by odor alone, which may be facilitated by signaler pheromones or an “odor print” [10]. Human infants have been shown to move toward the direction of breast odors, which indicates that some signals may release stereotyped behaviors [11]. It has been reported that body odors may change in response to changing emotions, which may then change the mood of exposed people by acting as mood-modulating pheromones [12]. In this regard, mood or emotion is known to affect the motivation to eat, food choice, eating speed, and amount of food intake [13], which suggests the possibility that a pheromone or chemosignal secreted due to an emotion may modulate appetite or eating behavior.

In line with the example of a modulator pheromone [12], a recent paper revealing that the sexual arousal and testosterone levels of men decrease in response to sniffing the sad tears of women is of particular interest [14]. Intriguingly, a functional magnetic resonance imaging study revealed that the sad tears of women regulated brain activity in the hypothalamus [14], where the appetite center is located [15]. Given that the chemosignals in sad tears can modify hypothalamic activity, we hypothesized that they may modulate appetite and eating behavior as well as sexual arousal. In this process, sad tears may affect emotion, which in turn may modulate appetite or eating behavior.

Normally, appetite decreases after the consumption of a meal through the complex interactions of neuronal, hormonal, and nutritional signals. Postprandial plasma contains increased levels of insulin, glucagon-like peptide-1 (GLP-1), cholecystokinin (CCK), and peptide YY (PYY) [16]. In addition to these satiety peptides, many nutrients and metabolites in the postprandial plasma may modulate appetite. Glucose and fatty acids can act on the central nervous system and regulate systemic glucose homeostasis [17]. In addition, recent metabolomics studies showed changes in metabolite profiles in the plasma after glucose or lipid challenges [18], [19]. Notably, the GLP-1 receptor [20] and the CCK receptor [21] have been detected in the mammalian olfactory system. In addition, intranasally administered exendin-4, a GLP-1 analogue, was reported to be actively transported to the olfactory bulb [22]. Although these peptides are unlikely to be volatile, non-volatile signals, such as peptides, could be detected by close sniffing [23], [24]. Taken together, despite the absence of firm evidence, we hypothesized that some unknown volatile metabolites as chemosignals or non-volatile components such as peptide hormones that are present in postprandial plasma may modulate appetite.

In this study, we conducted a randomized controlled crossover study examining the appetites and eating behaviors of men in response to sniffing the sad tears from female volunteers and their own fasting and postprandial plasma. To confirm whether we successfully induced a non-detectable or subliminal odor stimulus in our experimental condition, we measured serum testosterone levels, which was previously reported by Gelstein et al. [14].

## Subjects and Methods

### Subjects

Twenty young healthy male volunteers (aged 18–35 years) were recruited by a local advertisement. The inclusion criterion was a body mass index between 18.5 kg/m^2^ to 27 kg/m^2^. The exclusion criteria were diabetes mellitus or dyslipidemia, which were being treated with medication, and a past history of gastrointestinal surgery, except for an appendectomy or hemorrhoidectomy. Young, healthy female tear-donor volunteers (aged 18–35 years) were recruited by a local advertisement. They were screened for HIV, syphilis, hepatitis B and hepatitis C. Subjects with eyelid inflammation, conjunctivitis, keratitis, lacrimal duct obstruction, or skin diseases around the eyes were excluded. The study protocol was approved by the Institutional Review Board of the Seoul National University Hospital. This study is registered at ClinicalTrials.gov (NCT01389869). Written informed consent was obtained from each subject before any study-related procedure was performed. The study was conducted in compliance with the Declaration of Helsinki.

### Sad tear collection

Four healthy young female volunteers were enrolled. Two of the volunteers could not donate a sufficient amount of tears, thus we used tears from only two female donors (23 and 33 years of age). The tears were freshly prepared just before the experiment. The female tear donors arrived at 08:00 AM at the Biomedical Research Institute, Seoul National University Hospital. They washed their face with liquid soap (Baby Mild Liquid Soap; Dr. Bronner's, Escondido, CA), rinsed 10 times with tap water and removed the water with a paper towel (Yuhan-Kimberly Co., Seoul, Korea). Sad tears were collected in 1.8-ml Nunc CryoTubes (Thermo Fisher Scientific, Waltham, MA) at 1.0 cm below the margin of the lower eyelid while the donors watched sad documentary films (“Sarang” series; MBC production, Seoul, Korea) in an isolated room. In consideration of cultural characteristics, we used domestic films to maximize the intensity of the emotional response. The total volume of tears donated by 2 volunteers was approximately 2.5 ml per day. For the control experiments, 1.5 ml of normal saline was applied just below the lower eyelid and collected after trickling down a 1.0 cm distance. Handheld mirrors were provided to help in catching the tears. Afterwards, the tears were transferred for the sniffing experiments at room temperature.

### Study protocol

**Figure 1:**
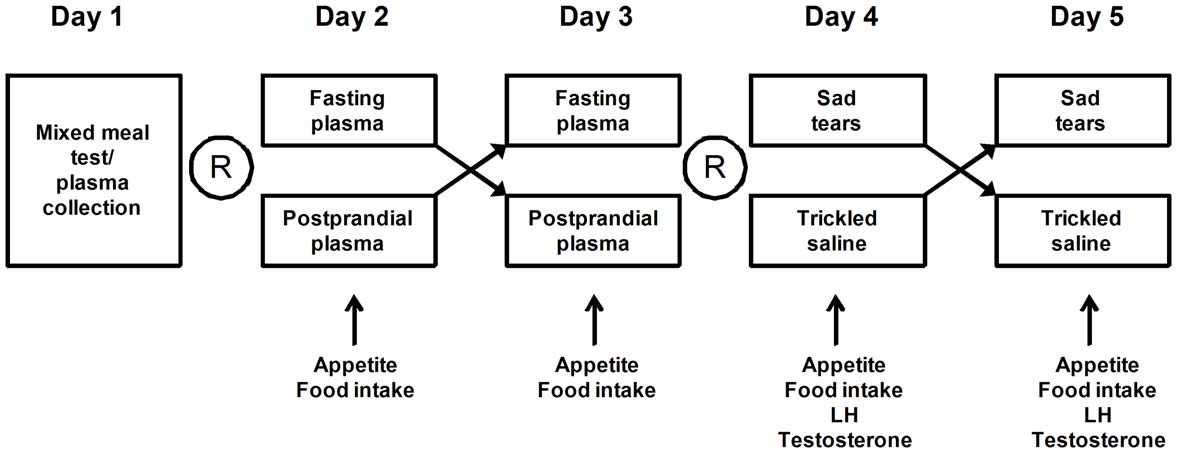
The study protocol. On day 1, we collected fasting and postprandial plasma samples before and during mixed meal tests in healthy male subjects (n = 20). On days 2 and 3, a sample of their own fasting or postprandial plasma at 60 min was randomly applied to the male subjects. On days 4 and 5, a sample of the fresh sad tears or the trickled saline from young healthy female volunteers was randomly applied to the male subjects. Details of the study protocol are described in the methods section. R denotes randomization.

Studies were performed over five consecutive days. Male volunteers were asked to not perform strenuous exercise or drink alcohol during the week before the study and during the study period. They visited the faculty's dining restaurant at Seoul National University Hospital after an overnight fast during the study period. The dining facility was only open for this experiment during the morning of the study. All studies began at 09:00 AM. On day 1, the height, weight, waist circumference, hip circumference, blood pressure, and pulse rate were measured by standard methods. An 18-gauge intravenous catheter was placed in the antecubital vein and flushed with 0.5 ml of heparinized saline (100 units per ml) for every blood draw. We instructed the volunteers to drink 2 cans of Jevity® (251 kcal/can with 35.1 g carbohydrate, 10.5 g protein, and 8.5 g fat per can; Abbott, Saint-Laurent, Québec, Canada) in 5 min. Blood samples were collected at 0, 15, 30, 60, 90, and 120 min after the consumption into a tube containing EDTA (BD Vacutainer, BD, Franklin Lakes, NJ) and a DPP4 inhibitor (Millipore, Billerica, MA). After centrifugation at 1500 g for 15 min at 4°C, the plasma were separated and stored at −80°C. Additional plasma samples for the sniffing experiments were collected at 0 min (fasting) and 60 min (postprandial) in EDTA tubes and stored at −20°C. We tested the effect of fasting vs. postprandial plasma on days 2 and 3 in a randomized order and a double-blind fashion. On days 4 and 5, we tested the effect of sad tears and trickled saline in a randomized order and a double-blind fashion. A randomization sheet was created by a staff from the Medical Research Collaborating Center, Seoul National University Hospital, who was not directly involved in this study. The randomization numbers were incorporated in the serial alphabets representing each experimental fluid (tears or saline or fasting or postprandial plasma). Because a staff from the Biomedical Research Institute, who was not directly involved in this study, distributed the samples to the volunteers, the researchers were blinded to the treatment. Olfactory discrimination tests were performed on days 2 and 4. Subjects were seated alone at a designated table and sniffed 5 tubes (1.5 ml; Eppendorf, Hamburg, Germany) containing 100 µl of fasting plasma and 5 tubes containing 100 µl of postprandial plasma on day 2 or 3 tubes containing 100 µl of tears and 7 tubes containing 100 µl of saline on day 4. They were told to identify the 5 tubes containing the fasting plasma and the 3 tubes containing the tears within 3 minutes. The study flow is depicted in Figure 1. One hundred microliters of the tears, saline, or fasting/postprandial plasma was applied to a band-aid (Nexcare 18 mm×73 mm; 3 M, St. Paul, MN), and then the band-aid was attached at the philtrum just below the nostrils. On days 2 and 3, the frozen plasma was thawed at room temperature with the cap closed and then used for the olfactory discrimination test and appetite test under a continuous exposure through sniffing. On days 4 and 5, freshly obtained sad tears were used for the olfactory discrimination test and appetite test. On days 2–5, we applied a band-aid that was folded backwards and contained either the plasma, tears, or saline to the philtrum at −10 min, as described above. At 0 min, we assessed appetite by a visual analog scale (VAS). Then, we measured the intake of the test food. For the test food, we provided an unrestricted amount of bread (Grain Baguette®; Paris Baguette Co., Seoul, Korea) and a fixed amount (200 ml) of milk (Seoul Milk Co., Seoul, Korea). We instructed the volunteers to eat the test meal until they were comfortably satisfied. The bread contains 231 kcal (69.4% from carbohydrate, 12.2% from protein, and 18.4% from fat) per 100 g, and the milk contains 70 kcal (29.4% from carbohydrate, 17.7% from protein, and 52.9% from fat) per 100 ml. We restricted milk consumption to avoid a decrease in the consumption of solid foods due to excessive liquid consumption. On days 4 and 5, blood samples were collected to measure the serum total testosterone and LH levels by a chemiluminescent immunoassay (Siemens Medical Solutions Diagnostics, Tarrytown, NY) at −10, 0, and 60 min. The amount of food was measured with a digital kitchen scale (KS-208CR; DRETEC, Saitama, Japan).

### Statistical analysis

All continuous variables were expressed as the means ± SEM. For the discrimination test, we used a χ^2^ test. Because the volunteers were instructed to select 5 tubes containing postprandial plasma out of the 10 tubes containing either fasting or postprandial plasma on day 2, the expected value for the correct discrimination was 2.5. Because the volunteers were instructed to select 3 tubes containing tears out of the 10 tubes containing either tears or saline on day 4, the expected value for the correct discrimination was 0.9. Since one volunteers failed to follow our instructions for the discrimination test, the df was 18. For comparisons of food intake, appetite, and changes of hormone levels, a Wilcoxon signed-rank test or an ANOVA was used. The within-subject comparison was performed by a χ^2^ test, which is explained in the figure legends in detail. A Spearman's correlation test was performed to examine the relationship between the appetite and food intake of the subjects. All statistical analyses were performed with GraphPad PRISM (GraphPad software, San Diego, CA). A *P*-value of less than 0.05 was considered statistically significant.

## Results

We recruited 20 healthy male volunteers, who were 26.3±4.6 years old and had a body mass index of 23.0±4.8 kg/m^2^. The fasting plasma glucose levels were 98.4±9.8 mg/dl and the 2-hr plasma glucose levels during the mixed meal tests were 109.2±21.3 mg/dl. The male volunteers could not discriminate the smell of fasting vs. postprandial plasma and sad tears vs. trickled saline (χ^2^ = 16.2, *P* = 0.422, df = 18 for sad tears and χ^2^ = 5.1, *P* = 0.999, df = 18 for postprandial plasma).

**Figure 2:**
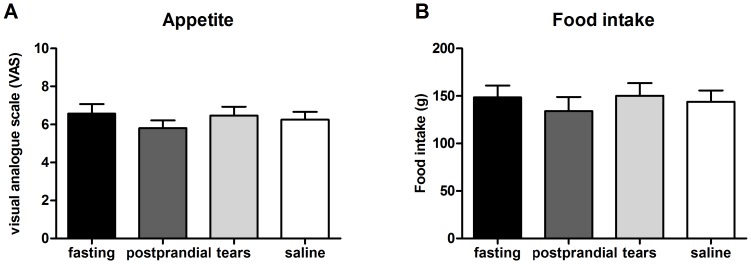
Comparison of appetite and food intake. A comparison of appetite, as measured by a VAS (A) and the amount of food intake (B), in response to sniffing samples of fasting or postprandial plasma, sad tears, or trickled saline.

**Figure 3:**
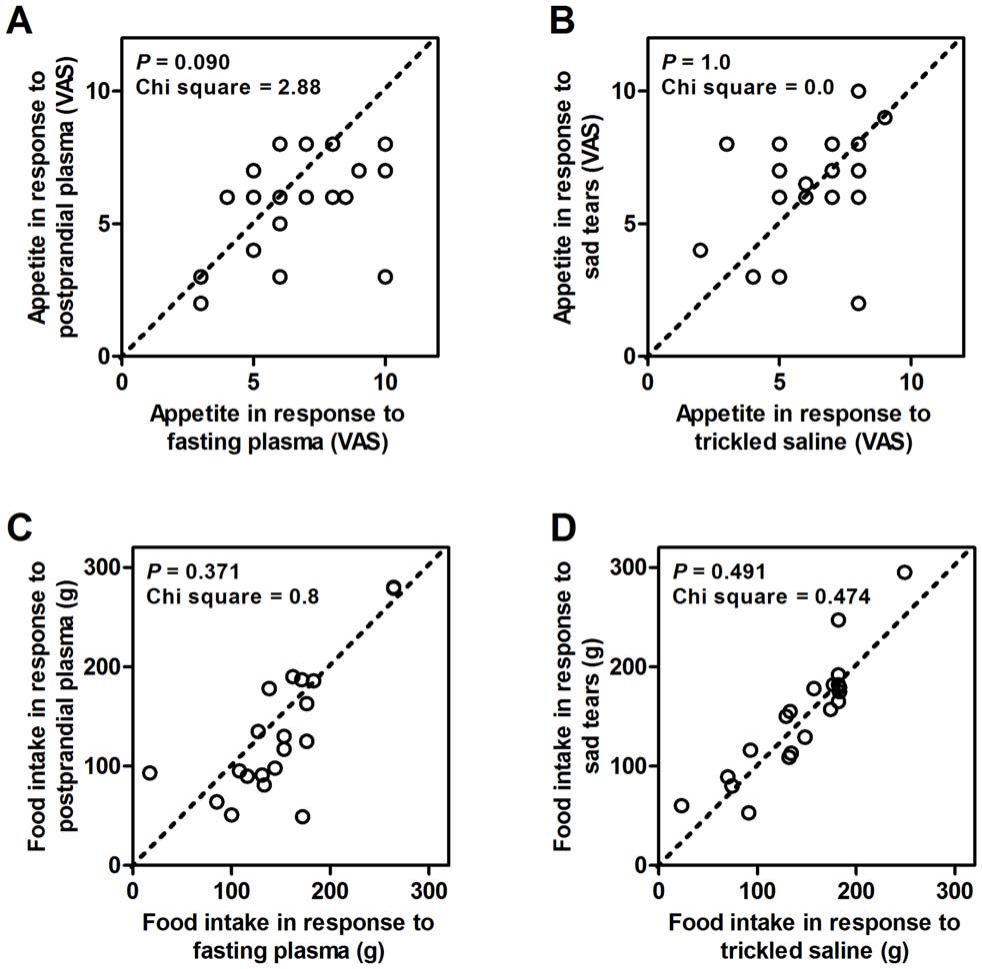
The within-subject comparisons for appetite and food intake. appetite measurements by a VAS with fasting vs. postprandial plasma (A); appetite measurements by a VAS with sad tears vs. trickled saline (B); the amount of food intake with fasting vs. postprandial plasma (C); and the amount of food intake with sad tears vs. trickled saline (D). Chi-square values and the corresponding *P*-values are shown. If the circles are on the diagonal line, there was no difference between the two conditions. If a greater number of circles are observed under the diagonal line, the values for the conditions shown in the horizontal axis were greater.

**Figure 4:**
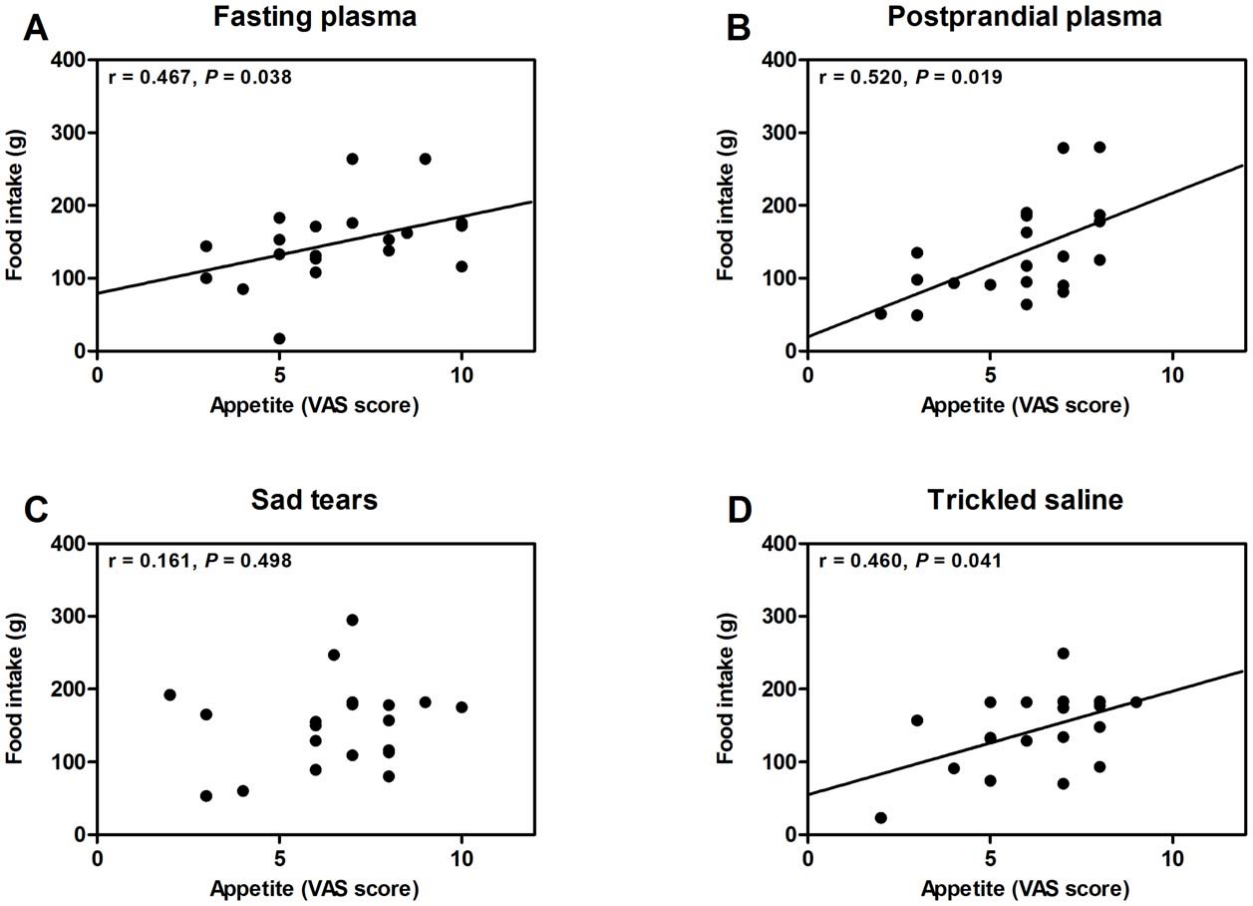
The correlation between appetite and food intake. Appetite was measured by a VAS. The correlation coefficients and corresponding *P*-values were calculated by a Spearman's test.

The appetite estimated by a VAS was comparable among the 4 groups (6.6±0.5 with fasting plasma, 5.8±0.4 with postprandial plasma, 6.5±0.5 with sad tears, and 6.3±0.4 with trickled saline; *P* = 0.674 by ANOVA) (Fig. 2A).The amounts of bread intake were also comparable among the 4 treatment groups (149±12 g with fasting plasma, 134±15 g with postprandial plasma, 150±13 g with sad tears, and 144±12 g with trickled saline; *P* = 0.758 by ANOVA) (Fig. 2B). There was no significant difference in the appetite and food intake according to the phase of the menstrual cycle of female volunteers (data not shown). For the within-subject comparisons, there were no differences in appetite in response to fasting plasma vs. postprandial plasma (Fig. 3A) or to the sad tears vs. the trickled saline (Fig. 3B). No within-subject differences were found for the comparisons of the food intake while sniffing fasting plasma vs. postprandial plasma (Fig. 3C) and sad tears vs. trickled saline (Fig. 3D). The total amount of calorie intake (bread and milk) showed a similar pattern (data not shown). The actual amounts of bread consumption were significantly correlated with the VAS score for appetite in the experiments with fasting plasma, postprandial plasma, and trickled saline (Fig. 4A, B, and D). However, this correlation was not observed in the experiment with sad tears (Fig. 4C).

**Figure 5:**
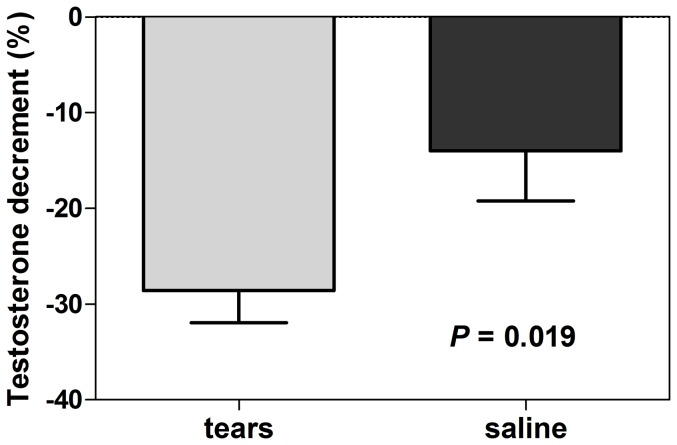
Serum testosterone. The percent decrease of serum testosterone levels from the baseline at −10 min with sad tears and trickled saline. The *P*-value was calculated by a Wilcoxon signed-rank test. With respect to the testosterone levels, 4 subjects were excluded from the analysis due to hemolytic samples.

On days 4 and 5, we measured the serum testosterone and LH levels. There was a greater percent decrease of serum testosterone levels at 60 min from the baseline at −10 min with the sad tears than with the trickled saline (−28.6±3.3% vs. −14.0±5.2%; *P* = 0.019; Fig. 5). There were no significant differences in the serum LH levels during the experiments (data not shown). In addition, there was no significant difference in the testosterone responses according to the phase of the menstrual cycle of female volunteers (data not shown).

## Discussion

We performed a randomized double-blind crossover study examining the effects of sad tears and fasting/postprandial plasma on appetite control and eating behavior. Consistent with a previous study [14], women's sad tears decreased the serum testosterone levels in men, which suggested that we successfully induced a non-detectable or subliminal odor stimulus in our experimental condition. However, neither sad tears nor postprandial plasma exhibited any effect on the appetite and food intake.

Similar to the findings by Gelstein et al. [14], we observed the decrease of serum testosterone in men who were exposed to women's sad tears, which might be mediated through chemosignals. Although only sparse evidence is available, human beings appear to have some pheromone-like chemosignals that function as primers [8], [9], signalers [10], releasers [11], and modulators [12], [14]. Aside from the question related to whether pheromones are present in humans, it is still unknown whether humans possess the apparatus to detect pheromone signals in the way that animals do. In mammals except for humans, pheromones are detected mainly by the vomeronasal organ (VNO) [25]. However, in humans, a VNO-like structure is present only during early fetal development and thereafter regresses to a vestigial organ [26]. Furthermore, most, but not all, of the genes considered as human counterparts for rodent pheromone receptors are pseudogenes that are not transcribed [27]. Intriguingly, the main olfactory system and the accessory olfactory systems, including the VNO, are partly overlapping, and the two systems converge in chemosignal processing [28]. Therefore, the sensing mechanism of human pheromone-like signals that have been reported may involve olfactory systems other than the VNO.

Gelstein et al. [14] demonstrated that the brain activity in the hypothalamus was unequivocally affected by sniffing sad tears. Although the appetite center is located in the hypothalamus [15], we could not observe any significant differences in the appetite (as measured by a VAS score) and the amount of test meal intake between the application of the sad tears and the trickled saline. Because the hypothalamus is composed of many nuclei with various functions, the areas showing differential activity in the previous study [14] may not be the appetite center. Additionally, one hypothalamic nucleus may have divergent function. For example, the arcuate nucleus, the appetite control center, contains Kisspeptin/Neurokinin B/Dynorphin (KNDy) cells, which regulate reproductive function [29]. In addition, melanocortin receptor 4 that is expressed in the hypothalamus is known to regulate not only food intake and energy expenditure but also sexual function [30]. These findings suggest that the hypothalamic region that controls reproductive function may also regulate appetite and eating behavior. We observed that the actual amounts of food consumption were significantly correlated with the VAS score for appetite in the experiments using the fasting plasma, postprandial plasma, and trickled saline, which is consistent with the previous report demonstrating a significant correlation between subjective appetite sensations measured by VAS and the amount of actual food intake [31]. However, there was no significant correlation between the VAS score for appetite and the actual amount of food intake in the experiment with sad tears. It is known that emotions affect eating behavior in a divergent fashion, with approximately 30% of people eating more and approximately 48% eating less [13]. In this regard, if sad tears modulate emotion-related appetite, the responses may also be divergent. However, we could not provide any firm evidence on this issue. Moreover, we assessed the appetite by a single VAS measurement rather than repeated measurements during the feeding experiment, which might not be enough to evaluate the repeat-reliability [32]. Further studies are necessary to examine whether sad tears may alter the appetite-eating behavior relation.

To the best of our knowledge, there is no evidence that human blood contains chemosignals to change behavior or biological functions by sniffing. Nevertheless, some unknown volatile metabolites as chemosignals or non-volatile components such as peptide hormones that are present in postprandial plasma may modulate appetite through the olfactory system. Although peptide hormones are not likely to be volatile, the movement of the lip during chewing may produce an aerosol that contains small peptides, and the aerosolized particles may be inhaled directly through the nostril by close sniffing. In this regard, it is very interesting that the GLP-1 receptor [20] and CCK receptor [21] are present in the olfactory system and intranasal administration of a GLP-1 analogue was demonstrated to be actively transported to the olfactory bulb [22]. However, we found that sniffing fasting and postprandial plasma had no effect on appetite and food intake in humans. Because we used frozen blood samples in this study, even though they were tightly sealed in a vial with a screw cap, some important volatile metabolites might have been lost during the freeze-thaw process. In addition, it is conceivable that aerosolized GLP-1 might be degraded by the action of local dipeptidyl peptidase-4 action in the nasal mucosa [33], resulting in a loss of its anorexigenic function. Further studies using fresh samples and/or a direct spray to the nasal mucosa rather than inhalation may be useful for elucidating a possible role of fasting and postprandial plasma on appetite regulation via the olfactory system.

In conclusion, sniffing human sad tears or postprandial plasma does not appear to have significant effect on appetite and food intake. However, further studies are needed to examine whether sad tears may alter the appetite-eating behavior relation.
